# A pilot study on aesthetic treatments performed by qualified aesthetic practitioners: efficacy on health-related quality of life in breast cancer patients

**DOI:** 10.1007/s11136-019-02133-9

**Published:** 2019-02-20

**Authors:** Serena Oliveri, Flavia Faccio, Silvia Pizzoli, Dario Monzani, Carolina Redaelli, Mirella Indino, Gabriella Pravettoni

**Affiliations:** 1 Department of Oncology and Hematoncology (DIPO), University of Milan, Via Festa del Perdono 7, 20122 Milan, Italy; 2 Applied Research Division for Cognitive and Psychological Science, IEO, Istituto Europeo di Oncologia IRCCS, via Ripamonti 435, 20141 Milan, Italy; 3 Dermophisiologique Oncology Aesthetics Center, IEO, Istituto Europeo di Oncologia IRCCS, via Ripamonti 435, 20141 Milan, Italy

**Keywords:** Skin toxicity, Aesthetic treatment, Cancer treatments, Quality of life, Health psychology

## Abstract

**Purpose:**

Cancer treatments often produce undesirable side-effects, such as skin toxicity, impacting on everyday functioning and health-related quality of life (HRQoL). This experimental study sought to determine whether aesthetic products and treatments could significantly decrease perceived skin symptoms, psychological distress and improve skin-related QoL (SRQoL).

**Methods:**

An experimental group composed of 100 breast patients was enrolled for specialized aesthetic treatments at the European Institute of Oncology (IEO) and compared to a control group of 70 breast patients who did not receive any aesthetic treatment. A measure of SRQoL (i.e., Skindex-16) and a distress thermometer were administered longitudinally at three time points: at baseline (T0), at 7 days from beginning of aesthetic treatment (T1) and at 28 days from beginning of aesthetic treatment (T2).

**Results:**

Results demonstrated the efficacy of aesthetic treatment in reducing distress and improving SRQoL: while the experimental group showed significant improvements in all HRQoL areas, the control group worsened. Specifically, at T1 and T2 there were significant improvements on distress and Skindex subscales in the experimental group, with an almost complete remission of perceived symptoms at T2. Moreover, all reported cutaneous reactions significantly improved after the specialized treatments, with no differences in SRQoL in skin reaction type.

**Conclusions:**

These findings demonstrate that aesthetic treatments for side-effects of cancer therapies can alleviate perceived distress and improve skin symptoms and HRQoL.

**Electronic supplementary material:**

The online version of this article (10.1007/s11136-019-02133-9) contains supplementary material, which is available to authorized users.

## Introduction

Invasive breast cancer is often treated with mastectomy or conservative surgery combined with radiotherapy (RT) or other cancer treatments, such as cytotoxic chemotherapy (CC) and targeted therapy (TT), depending on the nature and kind of breast cancer [1]. Side-effects of oncological therapies which are often overlooked are skin toxicity and skin-related disorders. Physicians tend to focus on treatment outcome and toxicity risk, failing to recognize the skin distress reported by the patient and creating a gap between patient’s perceived symptoms and those assessed by the physician [2]. Women receiving cancer therapy may experience skin-related side-effects that negatively affect not only adherence to therapy but even health-related quality of life (HRQoL) [3, 4]. For these reasons, preventing and managing skin reactions are becoming increasingly important to promote compliance with treatment, comfort and patient’s well-being [5–7].

The majority of the skin changes take several weeks from completion of therapy to heal [2]. For this reason, healing from skin-related disorders can be perceived as a relentless process due to worsening discomfort and increased skin changes [8]. For radiotherapy treatment, the first skin reactions appear within 1 to 4 weeks from the beginning of treatment, and the most unpleasant effects are those linked to tactile and nociceptive effects, such as pain, skin color and texture changes [9]. Chemotherapy can cause swelling, nail damage, and hand-foot syndrome, all of which impact on hand and feet functioning [10, 11]. Skin toxicity is also one of the main collateral effects of TT [12]. Although most patients report mild to moderate skin-related disorders, the level of distress and the impact on HRQoL is perceived as moderately high [2].

Overall, pain and skin-related disorders can be difficult to bear due to the visibility of skin changes and impact on day-to-day functioning. For these reasons, it is advisable to recommend the use of skin care products, such as aqueous creams, aloe vera, hyaluronic acid, which can improve self-image and subsequently decrease anxiety [13–19].

Although evidence regarding the efficacy for various products in the treatment of skin-related disorders is conflicting [16, 20], an important aspect of patient’s wellbeing, namely skin related QoL (SRQoL), is often neglected in the literature. Previous studies have investigated the effectiveness of aesthetic treatments in improving fatigue, anxiety, depression and sleep disturbances in various types of cancer at different stages [21–26]. However, SRQoL has rarely been introduced in previous studies; even if guidelines indicate that it should be incorporated to evaluate the efficacy of skin reaction management [14]. Schnur and colleagues [9] investigated SRQoL in women with stage 0–III breast cancer undergoing RT and noticed that women experienced emotional distress, physical discomfort, and body image disturbance. Changes in skin color increased anxiety, concern about others reactions and changes in their day-to-day functioning (e.g., changing bras and clothing). These changes impact emotionally on the patient’s perception of their body, as it gives a visible effect to cancer [27].

Summarizing, dermatological effects of cancer treatments have a negative impact on SRQoL through increased distress, withdrawal from relationships and increased risk of mood disturbances and non-adherence to treatment. The overall aim of the following study was to assess the efficacy of aesthetic treatments in promoting women’s perceived SRQoL. We hypothesize that aesthetic treatments performed by a specialized cosmetologist could significantly improve skin symptoms, patient’s psychological state and daily functioning reducing the negative impact of skin lesions on their HRQoL during cancer treatment.

## Materials and methods

### Participants

Participants in this study were breast cancer patients receiving CT, TT or radiotherapy at the European Institute of Oncology (IEO) between April 2016 and August 2017. A total of 100 patients were enrolled in the experimental group, with one drop-out and a total number of 99 participants included. Seventy patients were enrolled in the control group, with one drop-out and a total number of 69 participants included.

### Sampling procedure

Recruitment was performed by the oncologists specialized in dermatology or day hospital nurses working in the Dermatology Unit based on the characteristics of the skin injuries and compliance to therapy. Subjects who agreed to participate were assessed at the initial visit for adverse skin reactions and graded based on the NCI-CTCAEv3.0 (National Cancer Institute-Common Terminology Criteria for Adverse Events) [28] or RTOG/EORTC (Radiation Therapy Oncology Group/European Organization for Research and Treatment of Cancer) Radiation Toxicity Grading [29]. Only patients with grade I adverse skin reaction were selected to participate in this study. Patients who asked for aesthetic treatments were enrolled within the experimental group and referred to the Dermophisiologique Oncology Aesthetic Center for APEO dermatological treatments (*Professional Association of Oncological Aesthetics)*. Patients who did not ask for a professional aesthetic treatment were enrolled in the control group. The experimental group filled in the questionnaires during their visit to the Dermophisiologique Oncology Aesthetic Center, whereas the control group filled in the questionnaires soon after the medical visit for adverse skin reactions at T0 and was contacted via phone by APEO cosmetologists to complete their follow-up (T1 and T2). Higher grades of adverse skin reactions to therapies require medical intervention and were therefore excluded from enrollment (see Table 1 for a detailed description of inclusion and exclusion criteria).

**Table 1:** Inclusion and exclusion criteria for study enrollment

Inclusion criteria Diagnosis of breast cancer histologically confirmed Subjects treated with chemotherapeutic agents, targeted therapy or radiotherapy with skin side-effects Subjects with grade I of adverse skin symptoms Female subjects 18 of age Conditions favoring the correct execution of the proposed program Signature of informed consent
Exclusion criteria Other cancer diagnosis Pregnancy or breastfeeding in progress Subjects with adverse skin symptoms higher than grade I Psychic or other disorders Subjects with skin diseases, ulcers, dyschromia that can alter the accuracy of preexisting assessment or other skin conditions that do not allow application of the cosmetic product Known hypersensitivity or allergy to one of the components of the products

After 28 days from recruitment, the referring oncologists specialized in dermatology conducted a second evaluation on skin reactions to cancer treatments based on the NCI-CTCAEv3.0 or RTOG/EORTC scales.

### Sample size calculation

A sample size calculation was conducted using a repeated measure analysis of variance (ANOVA) within-between interaction (i.e., experimental vs. control group and time) approach with G*Power 3 statistical software with 5% of significance level, 80% of power, a weak correlation between measures of 0.3 and weak-medium effect size of 0.20. Because the assumption of sphericity may be unmet in our data, the required sample size was computed by applying a correction for a potential significant deviation from sphericity (ε = 0.5) [30]. This calculation showed that a total sample size of 94 was sufficient to detect a significant interaction effect between groups and time. This sample size is also sufficient to detect a significant main effect of intervention vs. control group (minimum required sample size = 108) and the significant main effect of time (minimum required sample size = 94).

### Measures

Patients of both experimental and control groups were administered the Skindex-16 scale [31] and the distress thermometer [32] at three time points: at recruitment (T0), after 1 week (T1), and after 28 days (T2) from enrollment.

The Skindex-16 is a 16-item self-report instrument that measures the effects of skin disease on HRQoL comprehensively. At the beginning of the questionnaire, the following statement is presented: “During the past week, how often have you been bothered by..”. Each question asks subjects to rate on a 7-point Likert scale (ranging from 0 “Never bothered” to 6 “Always bothered”) the level of concern or discomfort due to their specific skin condition (e.g., itching, burning, frustration about skin condition). It is composed of three subscales: perceived symptoms (items 1–5), emotions (items 6–11), and daily functions (items 12–16). Responses to each item were transformed into a linear scale of 100, varying from 0 (never bothered) to 100 (corresponding to 6, always bothered) [31]. Each raw score was then normalized for the statistical analysis. The final score is the average of the patient’s responses in a given domain (perceived symptoms, emotions, and daily functions). Higher scores indicate higher levels of discomfort or concern. This questionnaire is considered internally reliable (Cronbach α = 0.86–0.93) and has adequate test–retest reliability, construct, and content validity [33].

The distress thermometer is a one-item self-report screening tool, with a visual analogue scale ranging from 0 (no distress) to 10 (extreme distress) and a midpoint anchor labeled “moderate distress”, which is used to measure the level of psychological distress patients have experienced over the past week, including the day of screening completion. Cutoff scores have been validated and are considered of clinical utility in medical settings; moreover, overall scores have been compared to other well-known measures (i.e., Hospital Anxiety Depression Scale) to test construct validity [34, 35].

### Intervention

The experimental group received a 1-h session of specific aesthetics treatments protocols twice a week, by a qualified cosmetologist. The APEO cosmetologist is a professional dedicated to treatments that protect the skin from toxicity, solving blemishes, soothing and irritations due to cancer therapies. The APEO cosmetologists undergo a very rigid and certified training during which they acquire the scientific expertise to understand what cancer pathologies and therapies are, and how they act at the cutaneous level. The Professional Association of Oncological Aesthetics, in collaboration with oncologists, cancer researchers, plastic surgeons, and specialized cosmetologists lecturers, provides 120 h of training for qualified cosmetologists (3 years of professional school degree). Theoretical teaching is supported by a practical stage during which cosmetologists learn the protocols to be implemented based on cancer treatments skin toxicity and skin symptoms. They furthermore learn techniques of pedicure, manicure, and massage, specific for patients undergoing cancer therapy. After a final examination, the acquired competences on cancer aesthetic protocols are certified.

**Fig. 1:**
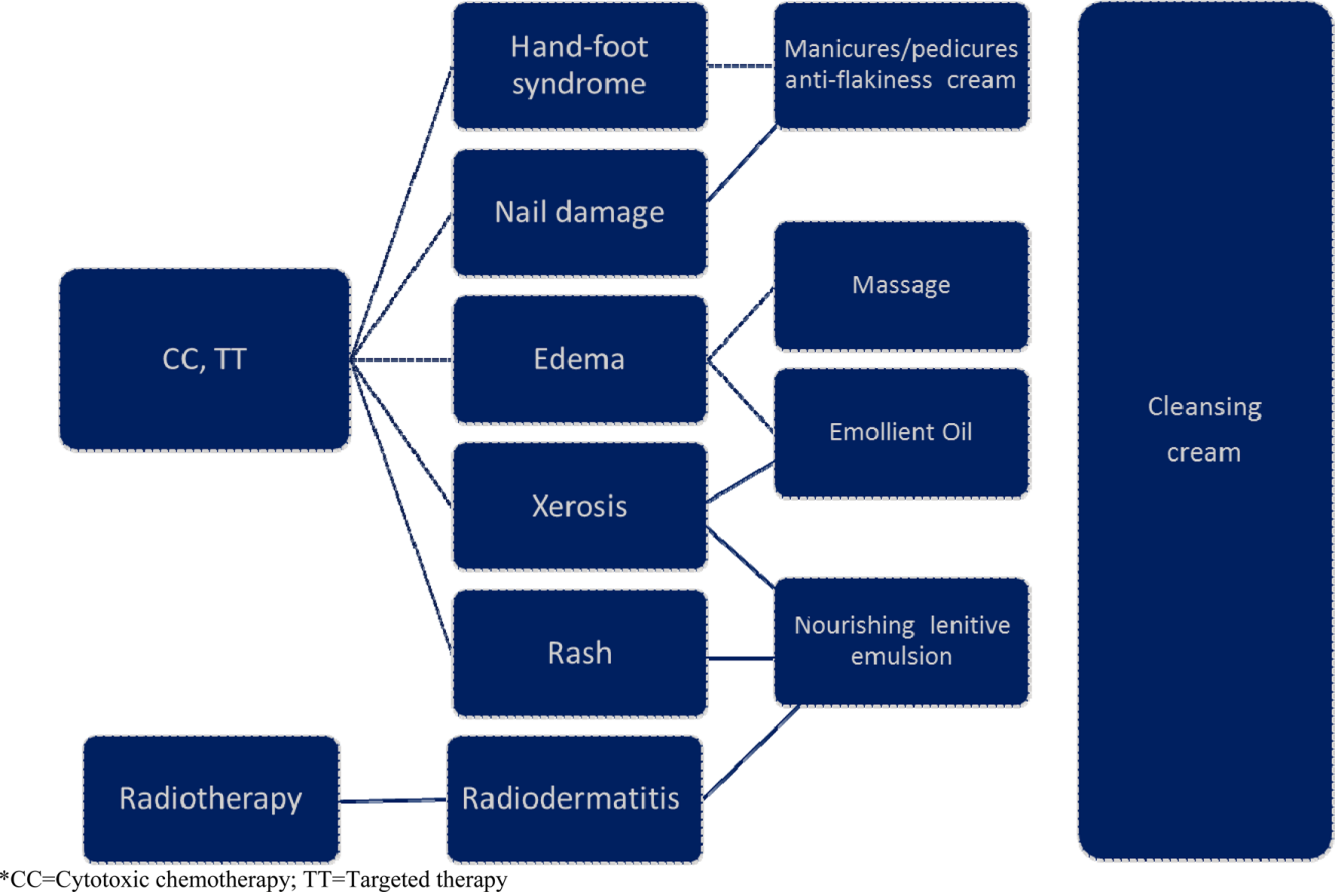
Cancer treatments, dermatological side-effects, corresponding wellness, and cosmetic treatments (*)

For this study, aesthetic treatments and cosmetic products were specifically created for skin care treatments during breast cancer therapies and provided by *APEO* cosmetologists. At T0, all patients’ lesions were evaluated, and specific cosmetic treatment protocols were applied (Fig. 1).

The type of aesthetic treatments is summarized in Supplementary Material 1, while treatment products composition (Ontherapy® by Dermophisiologique) and their properties are described in detail in Supplementary Material 2.

At T1, the patient underwent a second checkup with the APEO cosmetologist, for a second evaluation and a focused treatment. The same treatment was then applied at T2. Between T0, T1, and T2, the patient was instructed to apply specific cosmetic products daily, for cleansing, hydration, nourishment, and protection of their skin.

Cosmetic products and treatments provided by APEO cosmetologists have been endorsed by oncology physicians (the first oncological activities of the APEO were at the Dermophisiologique Oncology Aesthetic Center opened in the European Institute of Oncology IEO with the consent of prof. Umberto Veronesi, see https://www.ontherapy.it for more info about certifications), and the involvement of a medical professional was important for this study to ensure an objective medical evaluation of patients’ dermatological symptoms. APEO cosmetologist has certified competencies in evaluating the impact of grade I skin symptoms on patient’s HRQoL and can decide for appropriate personalized aesthetic protocols without the support/coordination of the oncology professional (that instead is required in case of higher adverse skin events). Nevertheless, aesthetic protocols are not part of standard medical care, and patients are free to use (or not) the Dermophisiologique products and the dermatological treatments object of the study without a medical supervision. All patients included in this study claimed to have correctly applied the cosmetic products. The simultaneous application of other cosmetics was strongly discouraged.

### Statistical analysis

Mixed analyses of covariance (ANCOVAs) were performed to evaluate the differences between experimental and control groups in the trend of Skindex total score (SRQoL), Skindex subscales and distress by verifying the interaction effect between time and presence/absence of aesthetic treatment. Because the control and the intervention groups differed at baseline in hand-foot syndrome and nail damage, these two variables were introduced as covariates in all these analyses. Furthermore, in the experimental group only, we applied a mixed ANOVA to verify the efficacy of APEO aesthetic treatments on SRQoL subscales and distress considering the different groups of cancer treatment patients (CC, TT, radiotherapy) by checking for the interaction effect between time and cancer treatment typologies. We performed post hoc comparisons by using the Bonferroni correction for multiple comparisons when significant results were obtained (*p* < 0.05).

At each time point, independent T tests were performed to verify significant differences in SRQoL total score and distress among patients in the experimental group based on the presence/absence of each specific dermatological side-effects (nail damage vs. no nail damage, hand-foot syndrome vs. no hand-foot syndrome, xerosis vs. no xerosis, etc.). We could not use ANOVA in this case since each patient usually had more than one dermatological side-effect due to cancer treatment.

All the analyses were performed with SPSS statistical software version 23.0.

## Results

Demographic characteristics, percentages of cancer treatments, clinical conditions, and dermatological side-effects at baseline are described in Table 2.

**Table 2:** Socio-demographic characteristics of the sample, patients’ cancer treatments, and dermatological side-effects

Variables	Experimental group		Control group		Statistic
	*N* = 99		*N* = 99		
Mean age (SD)	51.5 (10.8)		54.2 (12.0)		1.62^a^
**Cancer treatments**	*N*	%	*N*	%	
Cytotoxic chemotherapy	36	36.4	31	44.3	1.28^b^
Targeted therapy	31	31.3	21	30.0	
Radiotherapy	32	32.3	18	25.7	
**Cutaneous reactions**	* N*	%	*N*	%	
Hand-foot syndrome	37	37.4	16	22.9	4.01^b^*
Radiodermatitis	32	32.3	18	25.7	0.86^b^
Edema	31	31.3	14	20.0	2.68^b^
Xerosis	25	25.3	19	27.1	0.08^b^
Nail damage	14	14.1	27	38.6	13.31^b^**

*SD* standard deviation

*p < 0.05; **p < 0.01; *N* = number

^a^ t test; ^b^ Chi^2^

**Fig. 2:**
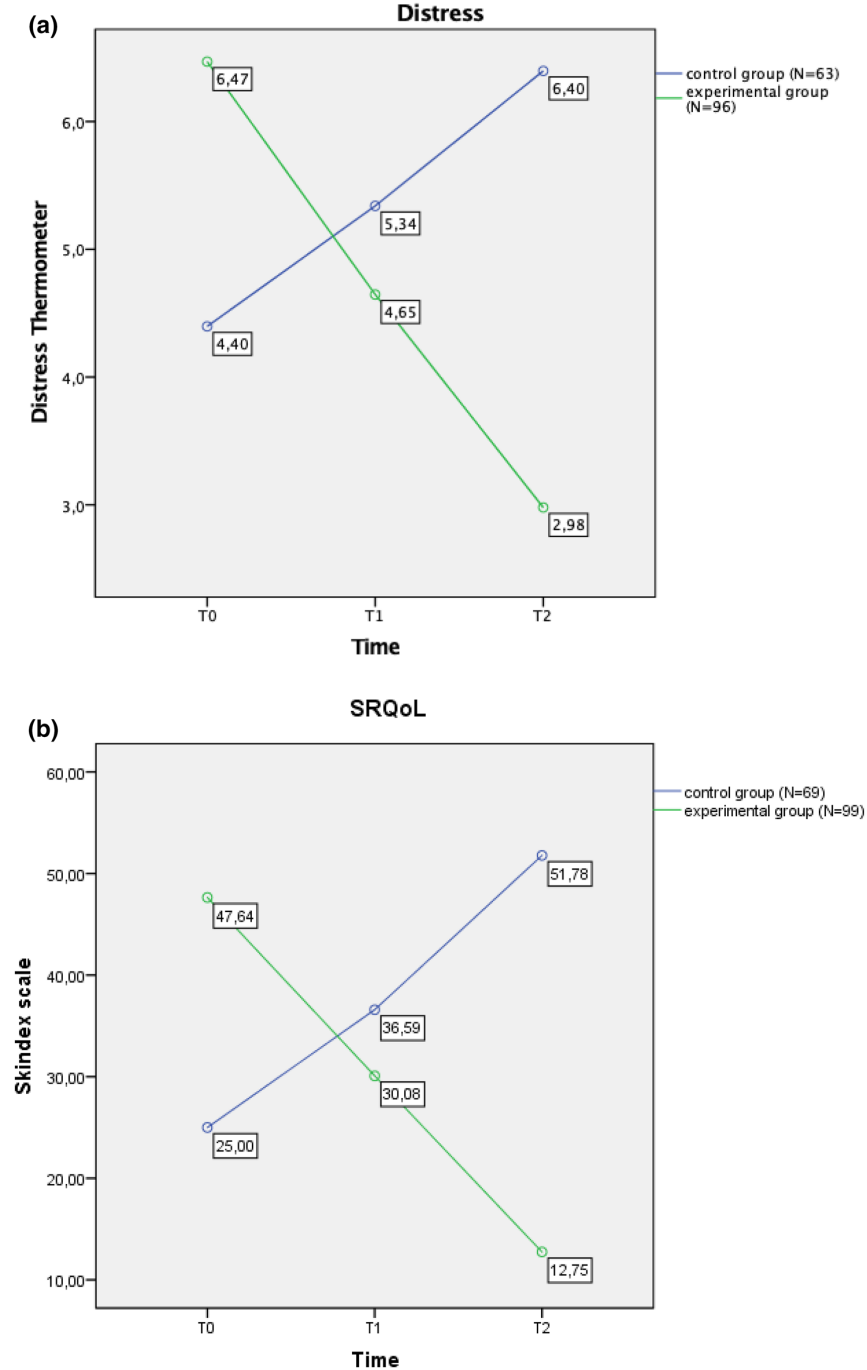
**a** Interaction between time and aesthetic treatment effects on distress levels in the experimental and control group; **b** interaction between time and aesthetic treatment effects on overall SRQoL in the experimental and control group. Mean scores of distress thermometer and Skindex-16 scale at T0, T1, and T2 are reported in the diagram labels

The mixed ANCOVA revealed that there was a significant interaction between time and presence/absence of aesthetics treatments on distress levels (*F*_2,290_ = 80.75; *p* < 0.001), with an opposite trend between the experimental and the control group (see Fig. 2a). Post hoc comparison with Bonferroni correction showed a constant decrease in distress in the experimental group and a constant increase in the control group. At T0, the experimental group was more distressed than patients in the control group, whereas at T2 the control group showed higher distress than women who underwent aesthetic treatments. The two groups did not differ in distress scores at T1.

With regard to the Skindex-16 total score (an overall mean score of SRQoL), results showed again an opposite trend for the experimental and the control group, with a significant interaction between time and presence/absence of aesthetics treatments (*F*_2,330_ = 210.42; *p* < 0.001) (see Fig. 2b). Post hoc comparison with Bonferroni correction showed constant improvement of SRQoL in the experimental group and constant worsening of SRQoL in the control group. At T0, the experimental group reported a worse SRQoL due to dermatological side effect, compared to the control group, whereas at T1 and T2 the control group showed higher scores at Skindex, thus reporting a decrease in SRQoL.

We then investigated whether there were differences between the experimental and control groups in the trends of the three subscales of the Skindex-16 (i.e., perceived symptoms, emotions, and functioning).

Results showed that the experimental group and the control group differed in trend of perceived symptoms (*F*_2,330_ = 211.03; *p* < 0.01), emotions (*F*_2,330_ = 160.89; *p* < 0.01), and functioning (*F*_2,330_ = 109.02; *p* < 0.01).

Post hoc comparison with Bonferroni correction highlighted the significant improvement in the three subscales of Skindex in the experimental group and a constant worsening in the control group. The experimental group reported worse perceived symptoms at T0, with a higher mean score in the Skindex subscale compared to the control group, and marked improvements at T1 and T2, compared to control group scores. Emotions and functioning scores were significantly higher for the experimental group at T0, similar to the control group at T1 and significantly lower at T2 compared to the control group. Mean scores of Skindex subscales in the experimental and control group across time are reported in Table 3.

**Table 3:** Skindex-16 subscales and distress mean scores in the experimental and control groups, at T0, T1 e T2

Subscale	T0 enrollment	T1 after 7 days	T2 after 28 days
	Mean (SD)	Mean (SD)	Mean (SD)
Symptoms EG	54.0 (25.7)	32.6 (20.4)	12.7 (13.5)
Symptoms CG	30.9 (26.1)	44.0 (23.2)	61.4 (25.5)
Emotions EG	54.6 (27.4)	35.1 (22.7)	15.2 (14.6)
Emotions CG	26.3 (30.1)	38.0 (26.8.0)	52.2 (28.6)
Functions EG	34.3 (26.7)	22.6 (21.0)	10.4 (13.7)
Functions CG	17.9 (26.3)	27.8 (24.9)	41.7 (29.3)

*EG* experimental group, *CG* control group, *SD* standard deviation

**Fig. 3:**
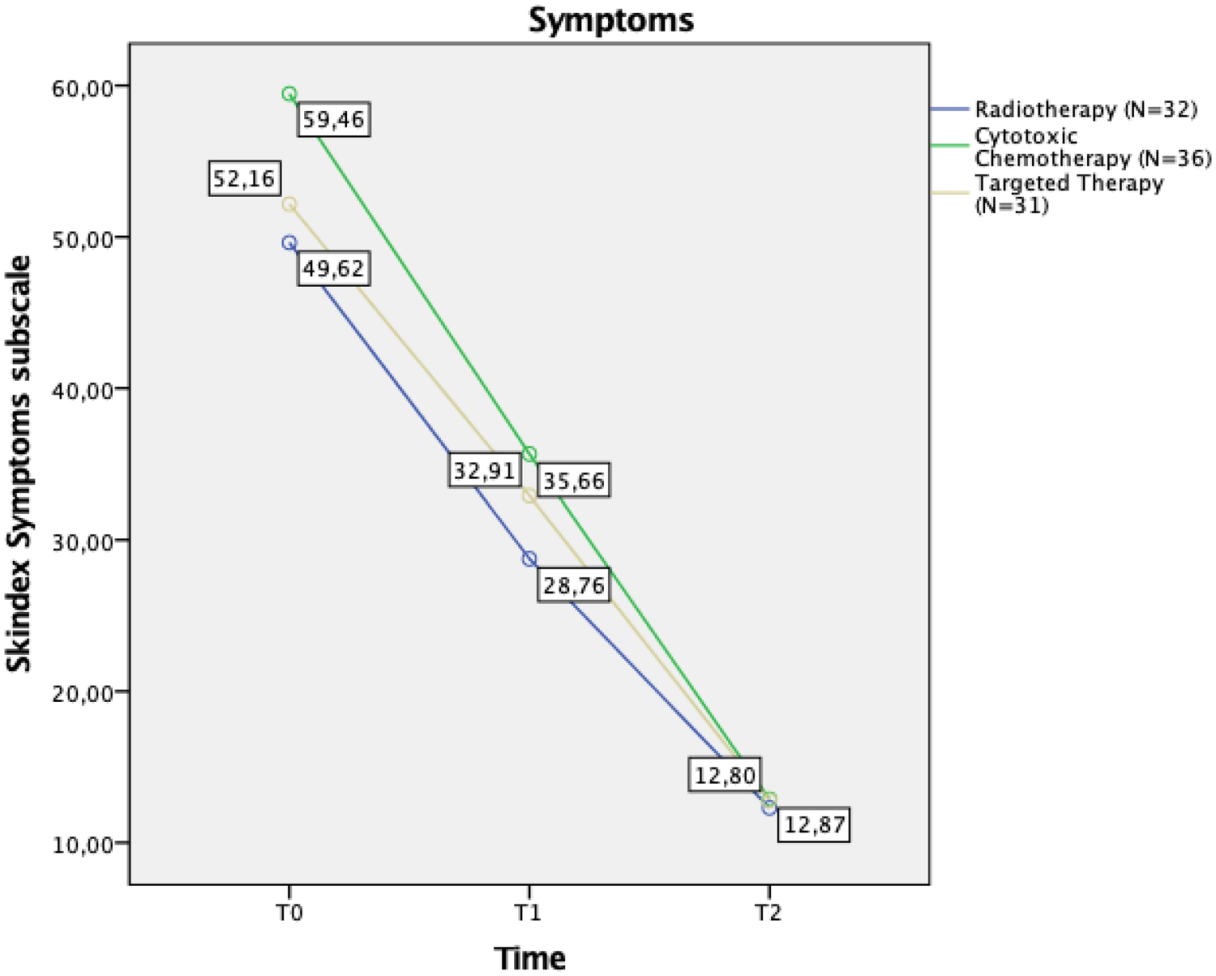
Interaction between time and aesthetic treatment effects on perceived symptoms in different cancer treatments groups. Mean scores of Skindex symptoms subscale at T0, T1, and T2 are reported in the diagram labels

We explored possible differences in Skindex subscales among patients in the experimental group, based on the different cancer treatments (CC, TT, radiotherapy) they were undergoing. Outcomes showed that there was no significant difference among groups in perceived dermatological symptoms (*F*_4,192_ = 1.282; *p* > *0.05*), with a similar trend during the period of their aesthetic treatment. They all had a significant improvement in perceived symptoms, with a clear reduction in the same after 28 days of aesthetic treatments (Fig. 3). Post hoc comparison with Bonferroni correction showed no differences among groups at T0, T1, and T2.

The same results emerged for the Emotion subscale (*F*_4,192_ = 0.78; *p* > 0.05), and post hoc showed no differences among groups at different time points.

**Fig. 4:**
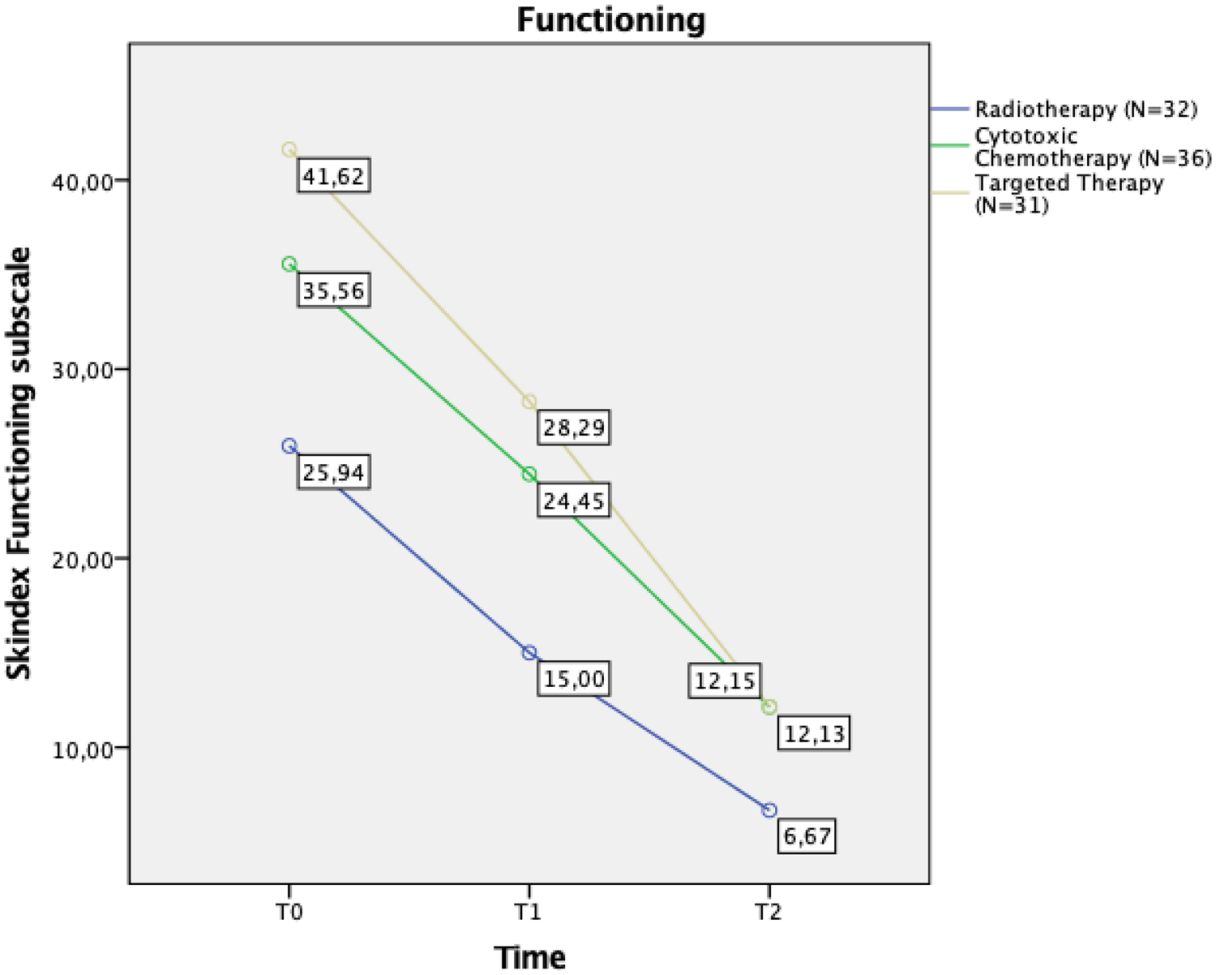
Interaction between time and aesthetic treatment effects on functioning in different cancer treatments groups. Mean scores of Skindex Functioning subscale at T0, T1, and T2 are reported in the diagram labels

Data showed no significant interaction between time and cancer treatment on functioning (*F*_4,192_ = 1.88; *p* > 0.05), showing that all the cancer treatment groups had an equal improvement in functioning after 1 week and at 28-day follow-up. Post hoc comparison with Bonferroni correction showed that patients who underwent TT were significantly more compromised in their functioning compared to patients who underwent radiotherapy at T0 and T1 (Fig. 4).

Results showed no significant differences in distress trends between patients undergoing CC, TT, and radiotherapy (*F*_4,186_ = 0.39; *p* > 0.05). Post hoc comparison showed all cancer treatment groups had comparable distress levels at each time point and all groups significantly improved over time.

Finally, a significant improvement in perceived SRQoL was registered for all cutaneous reactions to cancer treatments (Hand-foot syndrome, Radiodermatitis, Edema, etc.) after APEO aesthetic intervention. Differences emerged among patients with the hand-foot syndrome at T0, who had worse SRQoL than the group without this side-effect, and radiodermatitis at T1 and T2 (Table 4), who perceived less suffering than people with other cutaneous reactions to cancer treatments.

**Table 4:** Mean differences in Skindex-16 total scores (QoL) among patients with/without specific dermatological symptoms

Side-effects	*N*	T0	t	T1	t	T2	t
		Mean (SD)		Mean (SD)		Mean (SD)	
H-f syndrome Y	37	53.2 (21.3)	1.9*	34.1 (17.0)	1.7	13.7 (12.2)	0.6
H-f syndrome N	62	44.3 (21.3)		27.7 (17.1)		12.2 (11.7)	
Radioderm Y	32	41.2 (21.5)	− 2.1*	24.2 (14.9)	− 2.4*	10.5 (9.7)	− 1.3
Radioderm N	67	50.7 (21.2)		32.9 (17.7)		13.8 (12.6)	
Edema Y	31	52.9 (21.7)	1.6	33.6 (18.5)	1.4	12.5 (12.6)	− 0.1
Edema N	68	45.2 (21.4)		28.5 (16.6)		12.8 (11.6)	
Xerosis Y	25	47.8 (23.3)	0.4	31.5 (19.8)	0.5	16.2 (16.4)	1.7
Xerosis N	74	47.6 (21.2)		29.6 (16.4)		11.6 (9.7)	
Nail damage Y	14	42.6 (17.9)	0.9	27.3 (15.1)	0.6	9.4 (8.9)	1.1
Nail damage N	85	48.5 (22.2)		30.5 (17.6)		13.3 (12.2)	

*SD* standard deviation, *H-*f hand-foot; *Y* yes; *N* no

*p < 0.06

No significant differences emerged among patients in distress levels at T0, T1, and T2 based on dermatological symptoms.

The subjective evaluation of the patients in the experimental group, detected with the Skindex-16 scale and the Distress Thermometer, corresponded to a reduction in the level of symptoms and an improvement in functioning reported on the NCI-CTCAEv3.0 scale by the referring oncologist. In particular, patients who had the hand-foot syndrome, with difficulty in wearing shoes at T0, showed a reduction in rash and hyperkeratosis and had an enhancement in walking at T2. Patients with xerosis, who reported intense itching and initial break in the skin at T0, showed a reduction in desquamation and tingling at the end of the dermatological treatment.

Patients who were subjected to nail changes such as ridges (koilonychia), nail pitting, and erythema of the perionychium, due to cancer treatments, had difficulties in carrying out their daily activities at T0. They showed an improvement in the elasticity and compactness of the nail plate and a consistent reduction in erythema of the perionychium at T2.

Finally, patients who underwent radiotherapy and had grade 1 lesions evaluated with the RTOG / EORTC scale at T0, such as rash, itching, and initial skin atrophy, showed a reduction in erythema and itching and a blockage of the progression of skin atrophy at the end of the dermatological treatment (T2).

## Discussion

As highlighted previously, most of the available therapies are associated with side-effects that impact significantly on the patient’s HRQoL [36, 37]. Various studies reported that HRQoL during therapies decreases and that incidence of reported symptoms (e.g., fatigue, mood disturbances, skin, and sensation changes) increases [38–40]. Our data are in line with these studies, suggesting that patients’ evaluation of their HRQoL should always be taken into account and monitored during cancer treatments. Supportive cares, which focus on patients’ reported symptoms that are not measurable with laboratory tests or imaging procedures, may have a beneficial effect on overall patients’ well-being and adherence to cancer treatments.

The main evidence of the present study is that specialized APEO aesthetic treatments, conducted with specific cosmetic products (Ontherapy® by Dermophisiologique), are efficient in managing the impact that side-effects of CC, TT, and radiotherapy have on patients’ SRQoL and distress. Improvements in dermatological symptoms and functioning were also confirmed during the medical evaluation performed by the referring oncologists specialized in dermatology.

The APEO aesthetic treatments promote women’s psychological well-being and HRQoL, resulting in a clear reduction in perceived dermatological symptoms and an increase in positive emotions related to self-perception and relationship with others. In our samples, patients who asked for specialized aesthetic treatment and were enrolled in the experimental group perceived more accentuated dermatological symptoms compared to the control sample.

Even if dermatological side-effects caused psychological suffering, distress, and worse functioning in the experimental group, a week after the beginning of the aesthetic treatments significant improvements were registered in all domains of SRQoL and distress, with an almost complete healing of perceived skin symptoms, negative emotions, and negative functioning after 28 days of aesthetic treatments. In contrast, patients in the control group had significantly lower scores in all domains of SRQoL and their distress levels raised markedly, manifesting medium to high pain at 28 days follow-up. Other studies have reported advantages in applying cosmetic products on skin reactions due to oncological therapies [13, 14, 17], whereas Quintard and Lakdja [41] described the benefits of beauty treatments on psychological distress, body image and coping in breast cancer patients. Despite this evidence, currently, there are no standardized, recommended onco-aesthetics protocols to counteract the impact of cancer treatments’ side-effects on SRQoL.

Consistent with previous literature [42–44], cytotoxic chemotherapy and targeted therapies turned out to have a worse impact on daily functioning compared to radiotherapy in our experimental group. Despite this initial discrepancy, no significant differences emerged between oncological therapies in any of SRQoL domains after 28 days of specialized aesthetic treatments; rather all patients benefited equally from APEO treatments. Results confirm the effectiveness and importance of suggesting aesthetic treatment to patients undergoing different kinds of breast cancer treatments, as this can promote cancer patients’ well-being and HRQoL before, during, and after treatments and reduce distress. In our study, women who had radiodermatitis perceived themselves as being overall less compromised in their HRQoL, while patients with hand-foot syndrome considered themselves as highly affected by their dermatological condition before starting APEO treatments. This result confirms past literature, which highlighted that hand-foot syndrome is the symptom which brings more distress, suffering, and discontinuation of therapy due to a marked decrease in patient’s HRQOL [45].

Some limitations of the current study should be mentioned. First, the control group is smaller than the experimental group. Moreover, patients were not randomly assigned to the experimental or the control group, as we included in the experimental group all patients that asked for specialized aesthetic treatment. As a consequence, the two groups differed at T0 regarding their SRQoL and distress levels, with patients in the experimental group reporting more concern and discomfort about their skin conditions. However, this initial difference between experimental and control group did not influence results about SRQoL and distress trends across time. Further studies should replicate these findings with bigger samples and with diverse tumor diagnosis in randomized controlled trials, in order to promote the development of guidelines. Furthermore, the type of treated symptoms often overlapped. Thus, a comparison between specific symptoms groups was not possible: future research could investigate whether specific skin lesions are associated with worse SRQoL and psychological well-being.

## Conclusion

The efficacy of specialized aesthetic treatments on perceived distress and skin-related problems, highlighted in this study, demonstrates that they can become complementary therapies for cancer management. It is important to include psychological measures in studies assessing these skin injuries, as they often impact on their daily functioning and HRQoL. An integrative approach between oncology, psychology, and aesthetic skin management is strongly advised in order to offer the best comprehensive care to patients.

## Electronic supplementary material

Below is the link to the electronic supplementary material.

Supplementary material 1 (DOC 30 KB)

Supplementary material 2 (DOC 138 KB)
