# Supplementary material for: A pilot study on aesthetic treatments performed by qualified aesthetic practitioners: efficacy on health-related quality of life in breast cancer patients
Source: Qual Life Res. 2019 Feb 20;28(6):1543–53. doi: 10.1007/s11136-019-02133-9 (PMC6522456; doi:10.1007/s11136-019-02133-9)
Supplement: Supplementary file 1 — Supplementary material 1 (DOC 30 KB) [file 11136_2019_2133_MOESM1_ESM.doc]

Aesthetic treatments performed by APEO cosmetologist for side effects due to breast cancer treatments.

| **Dermatological symptoms** | **Aesthetic treatment** |
| --- | --- |
| Onycholysis and paronychia (Hand-foot syndrome) | Injuries such as onycholysis and paronychia are treated with specific manicures and pedicures treatments, with nail cut to cover the hyponychia and compress on the perionychium and nail plate, using the emollient oil and the anti-flakiness cream. The patient will daily use the cleansing cream and the anti-flakiness cream. |
| Radiodermatitis | Radiodermatitis are treated with 20 minutes of compress on the damaged area through the nourishing and lenitive emulsion, to reduce redness, restore the skin barrier and prevent patchy moist desquamation. The patient will daily use the cleansing cream and the nourishing and lenitive emulsion. |
| Hand-foot syndrome | The Hand-Foot Syndrome, characterized by skin breaks and hyperkeratosis, will require one hour of treatment. 10 minutes of use of compresses with the anti-flakiness emulsion followed by removal on the damaged skin and massage with the lenitive oil. The patient will use daily the cleansing cream to wash himself/herself and the anti-flakiness cream. In the case of Hand-Foot syndrome characterized by burning and erythema then the syndrome is treated with lenitive emulsion compresses. The patient will use on a daily basis the cleansing cream to wash himself/herself followed by the lenitive cream. |
| Xerosis | Xerosis will require one hour of treatment. In order to nourish the skin and to remove hyperkeratosis, 20 minutes of use of compresses with the emollient oil and the anti-flakiness cream are given, followed by a soft massage and an emollient oil application. |
| Edema | Edema is treated with a lymphatic massage using the emollient oil. The patient will use daily the cleansing cream to wash himself/herself followed by the lenitive and nourishing cream. |
