# Supplementary material for: A pilot study on aesthetic treatments performed by qualified aesthetic practitioners: efficacy on health-related quality of life in breast cancer patients
Source: Qual Life Res. 2019 Feb 20;28(6):1543–53. doi: 10.1007/s11136-019-02133-9 (PMC6522456; doi:10.1007/s11136-019-02133-9)
Supplement: Supplementary file 2 — Supplementary material 2 (DOC 138 KB) [file 11136_2019_2133_MOESM2_ESM.doc]

**DESCRIPTION OF COSMETIC PRODUCTS**

**NORMALIZING-PROTECTIVE CLEANSING CREAM**

**Description:**

Detergent emulsion that cleans the skin without altering its natural hydrolipic film thanks to the combination of lipids, surfactants and vegetal ingredients. Some of the natural substances that can be found are Chamomile extracts, Hamamelis Virginiana and Malva Sylvestris.

## *RECOMMENDED* as an *cosmetic adjuvant* for skin during oncological therapies. It can be used on the body and face when the skin is dry to extremely dry and presents redness, itchiness and flakiness.

| Therapeutic Properties | | | | | | | | |
| --- | --- | --- | --- | --- | --- | --- | --- | --- |
|  | *%* | Re-epithelizing | Anti-inflammatory | Antiseptic | Redness reduction | Regenerative | Hydrating | Emollient |
| *GLYCINE SOJA OIL* | 4% | + |  |  |  |  |  | + |
| *RICINUS COMMUNIS SEED OIL* | 4% |  |  |  |  |  |  | + |
| *HAMAMELIS VIRGINIANA*  *LEAF WATER* | 2,5% |  | + | + | + |  |  |  |
| *MALVA SYLVESTRIS LEAF WATER* | 2,5% |  | + | + | + |  |  |  |
| *CHAMOMILLA RECUTITIA*  *FLOWER EXTRACT* | 0,5% |  | + | + | + |  |  |  |
| *TOCOPHEROL* | 0,4% |  | + |  |  |  |  | + |

SOURCES: Roure (2012), Scarpa (1982), Graf (2000), Trüeb (2014), Gloor (2002), Razavi (2011), Yousefi (2010), Prudente (2013), Srivastava (2009), Singh (2011), Reiter (2007), Jiang (2014), Thiele (2005)

**FUNCTIONAL PROPERTIES**

**ACQUA HAMAMELIS VIRGINIANA:**

The distillate obtained from this plant has important anti-oxidant and anti-inflammatory properties.

**CHAMOMILE EXTRACT:**

It contains decongestant and anti-inflammatory properties. Moreover, it can accelerate the recovery process of skin lesions.

## **MALVA SYLVESTRIS:**

## It has an emollient, protective, refreshing effect on the skin and it reduces redness.

**ANTI-FLAKINESS CREAM WITH UREA 5%**

# **Description:**

Emulsion characterized by rich, nourishing properties specific to hands, feet and nails.

Thanks to its balanced formula with Jojoba Oil, rice and bran oil, hyaluronic acid and allantoin, it hydrates and soothes the skin. The percentage of urea used allows cellular turn over to take place and it is particularly recommended in cases of intensive treatment of extreme flakiness. It favors recreation of the stratum corneum and has an overall regenerative action.

## *RECOMMENDED as an cosmetic adjuvant for skin during oncological therapies. Useful on dry skin characterized by flakiness with itchiness and plaques.*

| Therapeutic Properties | | | | | | | | |
| --- | --- | --- | --- | --- | --- | --- | --- | --- |
|  | *%* | Re-epithelizing | Anti-inflammatory | Antiseptic | Redness reduction | Regenerative | Hydrating | Emollient |
| *UREA* | 5% |  |  |  |  | +++ | +++ |  |
| *SIMMONDSIA CHINENSIS SEED OIL* | 2% | + | + | + | + | + |  | + |
| *ORYZA SATIVA BRAN OIL* | 2% | + | + |  | + | + | + | + |
| *HYDROLYZED RICE PROTEIN* | 0,15% | + |  |  |  | + | + |  |
| *SODIUM HYALURONATE* | 0,15% | + |  |  |  |  | + |  |
| *ORYZA SATIVA STARCH* | 0,5% | + |  |  | + | + | + |  |
| *ALLANTOIN* | 0,7% | + | + |  | + | + | + |  |

SOURCES: Lòden (2003), Bissonnette (2010), Grether-Beck (2012), Habashy (2005), Pazyar (2013), Abdel-Mageed (2014), Heinemann (2008), Saenjum (2012), Fabian (2011), Avvantaggiato (2015), Weindl (2004), Price (2005), Necas (2008), Burlando (2014), Araújo (2010), Savić (2015)

**FUNCTIONAL PROPERTIES**

## **UREA:**

Molecule which can increase by 100% hydration of the stratum corneum. This molecule also has a regenerative action and removes dead skin cells.

**JALURONIC ACID AT HIGH MOLECULAR WEIGHT:**

Maintains skin hydrated, turgid, plastic and viscose.

## **RICE BRAN OIL:**

## Oil containing vitamin E, Y-oryzanol and phytosterol with emollient and nourishing properties.

## **RICE (MIXTURE):**

## Defends the skin from UVA rays and has a hydrating effect on the skin.

## **JOJOBA OIL:**

Oil rich of natural anti-oxidants, is an effective emollient product which gives elasticity to the skin. It is chemically very similar to human sebum.

## **ALLANTOINA:**

## Molecule that favors the rapid proliferation of cells and increases recovery of ulcers, burns, inflammatory states and injuries.

**EMOLLIENT OIL**

# **Description:**

Mixture of vegetal oils and butter (sweet almond oil, jojoba oil, sunflower seed oil) balanced in order to obtain an anti-oxidant and regenerative product for the skin. It increases the natural skin barriers and contrasts excessive skin dryness.

## *RECOMMENDED as a cosmetic adjuvant for the skin during oncological therapies. It should be used on dry to very dry skin, nails and area surrounding them. Can be applied on the body.*

| Therapeutic Properties | | | | | | | | |
| --- | --- | --- | --- | --- | --- | --- | --- | --- |
|  | *%* | Re-epithelizing | Anti-inflammatory | Antiseptic | Redness reduction | Regenerative | Hydrating | Emollient |
| *PRUNUS AMYGDALUS DULCIS OIL* | 67,24% | + | + |  | + |  |  | + |
| *SIMMONDSIA CHINENSIS SEED OIL* | 12% | + | + | + |  |  |  | + |
| *HELIANTHUS ANNUUS SEED OIL* | 7% |  |  |  | + |  |  | + |
| *GLYCINE SOJA OIL* | 7% | + |  |  |  |  |  | + |
| *BUTYROSPERMUM PARKII BUTTER* | 5% | + | + |  | + | + | + | + |
| *TOCOPHEROL* | 0,5% | + | + |  | + |  |  |  |
| *ASCORBYL PALMITATE* | 0,1% | + |  |  |  | + |  |  |

SOURCES: Ahmad (2010), Bährle-Rapp (2007), Habashy (2005), Pazyar (2013), Abdel-Mageed (2014), Kanti (2014), Bashir (2015), Roure (2012), Goreja (2004), Verma (2012), Reiter (2007), Jiang (2014), Thiele (2005), Üner (2005)

**FUNCTIONAL PROPERTIES**

## **ALMOND OIL:**

## Contains emollient, nourishing, dermo-protective and purifying and anti-itchiness properties.

## **OLIO DI JOJOBA**

Oil rich of natural anti-oxidants, is an effective emollient product which gives elasticity to the skin. It is chemically very similar to human sebum.

**SUNFLOWER SEED OIL:**

Contains mainly mono and poli fat acids and vitamin E. It has emollient and nourishing properties.

**NOURISHING AND LENITIVE EMULSION FOR REDNESS AND ITCHINESS**

# **Description**:

Emulsion oil in water with liquid-crystal technology, characterized by a rich and smooth texture. Enriched with vegetal extracts (black currant oil and 18β glycyrrhetinic acid) which have strong lenitive properties that contrast inflammatory states and reduce dramatically redness and itchiness. Vegetal lipids allow the skin barrier to be restored and protect the skin from dehydration. These also restore the immune system’s functioning.

*RECOMMENDED* *as a cosmetic adjuvant for the skin during oncological therapies. It should be used on dry to very dry skin, with redness and itchiness, on body and face.*

| Therapeutic Properties | | | | | | | | |
| --- | --- | --- | --- | --- | --- | --- | --- | --- |
|  | *%* | Re-epithelizing | Anti-inflammatory | Antiseptic | Redness reduction | Regenerative | Hydrating | Emollient |
| *GLYCINE SOJA OIL* | 6% | + |  |  |  |  |  | + |
| *BUTYROSPERMUM PARKII BUTTER* | 1,5% | + | + |  | + | + |  | + |
| *RIBES NIGRUM SEED OIL* | 0,75% |  | + | + | + |  |  | + |
| *TREHALOSE* | 0,7% | + |  |  |  | + | + |  |
| *ORYZA SATIVA BRAN OIL* | 0,5% |  | + |  | + |  | + | + |
| *UREA* | 0,65% |  |  |  |  | + | + |  |
| *SODIUM HYALURONATE* | 0,63% | + |  |  |  |  | + |  |
| *GLYCYRRHETINIC ACID* | 0,63% |  | + | + | + |  |  |  |
| *ALOE BARBADENSIS LEAF JUICE* | 0,5% | + | + | + | + |  |  |  |
| *TOCOPHEROL* | 0,46% | + | + |  | + |  |  |  |
| *OLEA EUROPEA OIL  UNSAPONIFIABLES* | 0,46% | + |  |  | + |  |  | + |
| *CERAMIDE 3* | 0,44% | + |  |  |  | + |  |  |
| *CERAMIDE 6 II* | 0,44% | + |  |  |  | + |  |  |
| *CERAMIDE 1* | 0,44% | + |  |  |  | + |  |  |
| *TOCOPHERYL ACETATE* | 0,4% | + | + |  | + |  |  | + |
| *PHYTOSPHINGOSINE* | 0,37% | + | + |  |  | + | + |  |
| *CHOLESTEROL* | 0,37% | + |  |  |  | + |  |  |
| *ASCORBYL PALMITATE* | 0,28% | + |  |  |  | + |  |  |

SOURCES: Roure (2012), Roure (2012), Goreja (2004), Verma (2012), Tabart (2012), Ghosh (2006), Linnama (2010), Luzardo (2000), Richards (2002), Burlando (2014), Lòden (2003), Bissonnette (2010), Grether-Beck (2012), Avvantaggiato (2015), Weindl (2004), Price (2005), Kao (2010), Ming (2013), Vibha (2009), Radha (2014), Fox (2014), Choi (2003), Reiter (2007), Jiang (2014), Thiele (2005), Koca (2011), Eidi (2012), Choi (2005), Coderch (2003), Mizutani (2009), Thiele (2007), Hoskin (2012), Kim (2006), Rawlings (2005), Pavicic (2007), Norlén (2001), Jungersted (2008), Feingold (2007), Jiang (2014), Thiele (2005), Üner (2005)

**FUNCTIONAL PROPERTIES**

**GLYCYRRHETINIC ACID:**

Contains anti-inflammatory, anti-allergic, anti-eczema properties and accelerate scar formation. These effects have been tested on injuries, ulcers and phlogosis.

**CERAMIDE 3, CERAMIDE 6, CERAMIDE 1:**

The application of these ceramides hydrates the skin and increases the protective barrier of the stratum corneum, making the skin less dry and sensitive.

**BLACK CURRANT OIL**:

Rich in anthocyanin and polyphenols, it has anti-oxidant, anti-thrombotic and anti-inflammatory properties.

**JALURONIC ACID AT HIGH MOLECULAR WEIGHT:**

Maintains skin hydrated, turgid, plastic and viscose.

**SHEA BUTTER**:

Butter with emollient, lenitive, hydrating and re-epithelizing properties. High in fat acids and rich in vitamin A, E and F which foster hydration and skin elasticity.

**UREA:**

Allows for cellular turn over to occur and it is particularly recommended in intensive treatment of extreme peeling.

**UNSAPONIFIABLE FRACTION OF OLIVE OIL**:

The non-triglyceride fraction of olive oil is rich in phytosterol and triterpene alcohols with emollient, nourishing, sebum-balancing properties.

## **TOCOPHERYL ACETATE**:

## Contains vitamin E and has an anti-inflammatory and lenitive action on the skin.

## **ALOE BARBADENSIS**:

## Extract containing big quantities of mucilage, but also lipids, enzymes, vitamins, minerals, glycol-proteins. It has anti-ageing and emollient properties.

**References**

# Abdel-Mageed, Wael M., et al. "Simmondsia chinensis: a rich source of bioactive flavonoids and lignans." *Industrial Crops and Products* 60 (2014): 99-103.

Ahmad, Zeeshan. "The uses and properties of almond oil." *Complementary therapies in clinical practice* 16.1 (2010): 10-12.

Araújo, Lorena Ulhôa, et al. "Profile of wound healing process induced by allantoin." *Acta cirurgica brasileira* 25.5 (2010): 460-461.

Avantaggiato, A., et al. "HYALURONIC ACID: THE USE OF ITS PRECURSOR IN SKIN BIO-STIMULATION." *Journal of biological regulators and homeostatic agents* 29.3 (2015): 647-654.

Bashir, Tasneem, et al. "Chemistry, pharmacology and ethnomedicinal uses of Helianthus annuus (Sunflower): a review." *Pure and Applied Biology* 4.2 (2015): 226.

Bissonnette, Robert, et al. "A double‐blind study of tolerance and efficacy of a new urea‐containing moisturizer in patients with atopic dermatitis." *Journal of cosmetic dermatology* 9.1 (2010): 16-21.

Burlando, Bruno, and Laura Cornara. "Therapeutic properties of rice constituents and derivatives (Oryza sativa L.): a review update." *Trends in food science & technology* 40.1 (2014): 82-98.

Choi, Seongwon, and Myung-Hee Chung. "A review on the relationship between Aloe vera components and their biologic effects." *Seminars in integrative medicine*. Vol. 1. No. 1. WB Saunders, 2003.

Choi, Myeong Jun, and Howard I. Maibach. "Role of ceramides in barrier function of healthy and diseased skin." *American journal of clinical dermatology* 6.4 (2005): 215-223.

Coderch, Luisa, et al. "Ceramides and skin function." *American journal of clinical dermatology* 4.2 (2003): 107-129.

Eidi, Akram, et al. "Antinociceptive and anti-inflammatory effects of olive oil (Olea europeae L.) in mice." *Pharmaceutical biology* 50.3 (2012): 332-337.

Fabian, Cynthia, and Yi-Hsu Ju. "A review on rice bran protein: its properties and extraction methods." *Critical reviews in food science and nutrition* 51.9 (2011): 816-827.

Feingold, Kenneth R. "Thematic review series: skin lipids. The role of epidermal lipids in cutaneous permeability barrier homeostasis." *Journal of lipid research* 48.12 (2007): 2531-2546.

Fox, Lizelle T., et al. "In vivo skin hydration and anti-erythema effects of Aloe vera, Aloe ferox and Aloe marlothii gel materials after single and multiple applications." *Pharmacognosy magazine* 10.Suppl 2 (2014): S392.

Ghosh, Dilip, et al. "Effects of anthocyanins and other phenolics of boysenberry and blackcurrant as inhibitors of oxidative stress and damage to cellular DNA in SH‐SY5Y and HL‐60 cells." *Journal of the Science of Food and Agriculture* 86.5 (2006): 678-686.

Gloor, M., et al. "Antiseptic effect of a topical dermatological formulation that contains Hamamelis distillate and urea." *Complementary Medicine Research* 9.3 (2002): 153-159.

Goreja, W. G. *Shea Butter: The Nourishing Properties of Africa's Best-Kept Natural Beauty Secret*. TNC International Inc, 2004.

Graf, J. "Herbal anti-inflammatory agents for skin disease." *Skin Therapy Lett* 5.4 (2000): 3-5.

Grether-Beck, Susanne, et al. "Urea uptake enhances barrier function and antimicrobial defense in humans by regulating epidermal gene expression." *Journal of Investigative Dermatology* 132.6 (2012): 1561-1572.

Habashy, Ramy R., et al. "Anti-inflammatory effects of jojoba liquid wax in experimental models." *Pharmacological research* 51.2 (2005): 95-105.

Heinemann, Riana JB, et al. "Tocopherols, tocotrienols, and γ-oryzanol contents in japonica and indica subspecies of rice (Oryza sativa L.) cultivated in Brazil." *Cereal Chemistry* 85.2 (2008): 243-247.

Hoskins, Aimee, et al. "Natural‐source d‐α‐tocopheryl acetate inhibits oxidant stress and modulates atopic asthma in humans in vivo." *Allergy* 67.5 (2012): 676-682.

Jiang, Qing. "Natural forms of vitamin E: metabolism, antioxidant, and anti-inflammatory activities and their role in disease prevention and therapy." *Free Radical Biology and Medicine* 72 (2014): 76-90.

Jungersted, Jakob Mutanu, et al. "Lipids and skin barrier function–a clinical perspective." *Contact dermatitis* 58.5 (2008): 255-262.

Kanti, Varvara, et al. "Influence of sunflower seed oil on the skin barrier function of preterm infants: a randomized controlled trial." *Dermatology* 229.3 (2014): 230-239.

Kao, Tzu-Chien, Ming-Huan Shyu, and Gow-Chin Yen. "Glycyrrhizic acid and 18β-glycyrrhetinic acid inhibit inflammation via PI3K/Akt/GSK3β signaling and glucocorticoid receptor activation." *Journal of agricultural and food chemistry* 58.15 (2010): 8623-8629.

Kim, Sujong, et al. "Phytosphingosine stimulates the differentiation of human keratinocytes and inhibits TPA-induced inflammatory epidermal hyperplasia in hairless mouse skin." *Molecular medicine* 12.1-3 (2006): 17.

Koca, Ufuk, et al. "Wound repair potential of Olea europaea L. leaf extracts revealed by in vivo experimental models and comparative evaluation of the extracts' antioxidant activity." *Journal of medicinal food* 14.1-2 (2011): 140-146.

Lodén, Marie. "Role of topical emollients and moisturizers in the treatment of dry skin barrier disorders." *American journal of clinical dermatology* 4.11 (2003): 771-788.

Luzardo, M. del C., et al. "Effect of trehalose and sucrose on the hydration and dipole potential of lipid bilayers." *Biophysical Journal* 78.5 (2000): 2452-2458.

Ming, Lee Jia, and A. C. Yin. "Therapeutic effects of glycyrrhizic acid." *Natural product communications* 8.3 (2013): 415-418.

Mizutani, Yukiko, et al. "Ceramide biosynthesis in keratinocyte and its role in skin function." *Biochimie* 91.6 (2009): 784-790.

Necas, J., et al. "Hyaluronic acid (hyaluronan): a review." *Veterinarni medicina* 53.8 (2008): 397-411.

Norlén, Lars. "Skin barrier structure and function: the single gel phase model." *Journal of investigative dermatology* 117.4 (2001): 830-836.

Pavicic, T., et al. "Anti‐microbial and‐inflammatory activity and efficacy of phytosphingosine: an in vitro and in vivo study addressing acne vulgaris." *International journal of cosmetic science* 29.3 (2007): 181-190.

Pazyar, N., et al. "Jojoba in dermatology: a succinct review." *G Ital Dermatol Venereol* 148.6 (2013): 687-691.

Price, Richard D., et al. "The role of hyaluronic acid in wound healing." *American journal of clinical dermatology* 6.6 (2005): 393-402.

Prudente, Arthur S., et al. "Pre-clinical anti-inflammatory aspects of a cuisine and medicinal millennial herb: Malva sylvestris L." *Food and chemical toxicology* 58 (2013): 324-331.

Radha, Maharjan H., and Nampoothiri P. Laxmipriya. "Evaluation of biological properties and clinical effectiveness of Aloe vera: A systematic review." *Journal of traditional and complementary medicine* 5.1 (2015): 21-26.

Rawlings, Anthony V., and Paul J. Matts. "Stratum corneum moisturization at the molecular level: an update in relation to the dry skin cycle." *Journal of Investigative Dermatology* 124.6 (2005): 1099-1110.

Razavi, Seyed Mehdi, et al. "Bioactivity of Malva sylvestris L., a medicinal plant from Iran." *Iranian journal of basic medical sciences* 14.6 (2011): 574.

Reiter, Elke, Qing Jiang, and Stephan Christen. "Anti-inflammatory properties of α-and γ-tocopherol." *Molecular aspects of medicine* 28.5 (2007): 668-691.

Richards, A. B., et al. "Trehalose: a review of properties, history of use and human tolerance, and results of multiple safety studies." *Food and Chemical Toxicology* 40.7 (2002): 871-898.

Roure, Romain, et al. "Methods to assess the protective efficacy of emollients against climatic and chemical aggressors." *Dermatology research and practice* 2012 (2012).

Saenjum, Chalermpong, et al. "Antioxidant and anti-inflammatory activities of gamma-oryzanol rich extracts from Thai purple rice bran." *Journal of Medicinal Plants Research* 6.6 (2012): 1070-1077.

Savić, Vesna Lj, et al. "Comparative study of the biological activity of allantoin and aqueous extract of the comfrey root." *Phytotherapy Research* 29.8 (2015): 1117-1122.

Scarpa, Antonio, and Antonio Guerci. "Various uses of the castor oil plant (Ricinus communis L.) a review." *Journal of ethnopharmacology* 5.2 (1982): 117-137.

Singh, Ompal, et al. "Chamomile (Matricaria chamomilla L.): an overview." *Pharmacognosy reviews* 5.9 (2011): 82.

Srivastava, Janmejai K., Mitali Pandey, and Sanjay Gupta. "Chamomile, a novel and selective COX-2 inhibitor with anti-inflammatory activity." *Life sciences* 85.19 (2009): 663-669.

Tabart, Jessica, et al. "Antioxidant and anti-inflammatory activities of Ribes nigrum extracts." *Food Chemistry* 131.4 (2012): 1116-1122.

Thiele, Jens J., Sherry N. Hsieh, and Swarna Ekanayake‐Mudiyanselage. "Vitamin E: critical review of its current use in cosmetic and clinical dermatology." *Dermatologic surgery* 31.s1 (2005): 805-813.

Thiele, Jens J., and Swarna Ekanayake-Mudiyanselage. "Vitamin E in human skin: organ-specific physiology and considerations for its use in dermatology." *Molecular aspects of medicine* 28.5 (2007): 646-667.

Trüeb, Ralph M. "North American virginian witch hazel (hamamelis virginiana): based scalp care and protection for sensitive scalp, red scalp, and scalp burn-out." *International journal of trichology* 6.3 (2014): 100.

Üner, M., et al. "Skin moisturizing effect and skin penetration of ascorbyl palmitate entrapped in solid lipid nanoparticles (SLN) and nanostructured lipid carriers (NLC) incorporated into hydrogel." *Die Pharmazie-An International Journal of Pharmaceutical Sciences* 60.10 (2005): 751-755.

Verma, Nandini, et al. "Anti-inflammatory effects of shea butter through inhibition of iNOS, COX-2, and cytokines via the NF-kB pathway in LPS-activated J774 macrophage cells." *Journal of Complementary and Integrative Medicine* 9.1 (2012): 4.

Vibha, J., et al. "A study on pharmacokinetics and therapeutic efficacy of Glycyrrhiza glabra: a miracle medicinal herb." *Botany Research International* 2.3 (2009): 157-163.

Weindl, G., et al. "Hyaluronic acid in the treatment and prevention of skin diseases: molecular biological, pharmaceutical and clinical aspects." *Skin Pharmacology and Physiology* 17.5 (2004): 207-213.

Yousefi, Maryam. "Malva sylvestris in the treatment of hand eczema." *Iranian Journal of Dermatology* 13.4 (2010): 131-4.

# Test in vitro: Valutazione in vitro dell’attività anti-infiammatoria di un prodotto cosmetico su colture cellulari. Test di efficacia BioBasic Europe – settembre 2016
